# Case Report: Consistent disease manifestations with a staggered time course in two identical twins affected by adenosine deaminase 2 deficiency

**DOI:** 10.3389/fimmu.2022.910021

**Published:** 2022-09-29

**Authors:** Federica Barzaghi, Maria Pia Cicalese, Matteo Zoccolillo, Immacolata Brigida, Matteo Barcella, Ivan Merelli, Claudia Sartirana, Monica Zanussi, Valeria Calbi, Maria Ester Bernardo, Francesca Tucci, Maddalena Migliavacca, Fabio Giglio, Matteo Doglio, Daniele Canarutto, Francesca Ferrua, Giulia Consiglieri, Giulia Prunotto, Francesco Saettini, Sonia Bonanomi, Patrizia Rovere-Querini, Giulia Di Colo, Tatiana Jofra, Georgia Fousteri, Federica Penco, Marco Gattorno, Michael S. Hershfield, Lucia Bongiovanni, Maurilio Ponzoni, Sarah Marktel, Raffaella Milani, Jacopo Peccatori, Fabio Ciceri, Alessandra Mortellaro, Alessandro Aiuti

**Affiliations:** ^1^ Pediatric Immunohematology and Bone Marrow Transplantation Unit, IRCCS San Raffaele Scientific Institute, Milan, Italy; ^2^ San Raffaele Telethon Institute for Gene Therapy, IRCCS San Raffaele Scientific Institute, Milan, Italy; ^3^ Vita-Salute San Raffaele University, Milan, Italy; ^4^ Institute for Biomedical Technologies, National Research Council, Segrate, Italy; ^5^ Clinical Genomics-Molecular Genetics Service, Istituto di Ricovero e Cura a Carattere Scientifico (IRCCS) Policlinico San Donato, San Raffaele Hospital, Milan, Italy; ^6^ Hematology and Bone Marrow Transplantation Unit, IRCCS San Raffaele Scientific Institute, Milano, Italy; ^7^ Bone Marrow Transplantation Unit, Pediatric Department of Milano-Bicocca University, Monza e Brianza per il Bambino e la sua Mamma Foundation, Monza, Italy; ^8^ Internal Medicine, Diabetes, and Endocrinology Unit, IRCCS San Raffaele Scientific Institute, Milan, Italy; ^9^ Immunology, Rheumatology, Allergy and Rare Disease Unit, IRCCS San Rafaelle Hospital, Milan, Italy; ^10^ Diabetes Research Institute, IRCCS San Raffaele Scientific Institute, Milan, Italy; ^11^ Clinica Pediatrica – Reumatologia e Centro Malattie Autoinfiammatorie, IRCCS Giannina Gaslini, Genova, Italy; ^12^ Department of Medicine and Biochemistry, Duke University Medical Center, Durham, NC, United States; ^13^ Pathology Unit, IRCCS San Raffaele Scientific Institute, Milan, Italy; ^14^ Immunohematology and Transfusion Medicine Unit, IRCCS Ospedale San Raffaele, Milan, Italy

**Keywords:** ADA2, adenosine deaminase 2 deficiency, neutropenia, T large granular lymphocytes, TLGL, HSCT

## Abstract

Deficiency of adenosine deaminase 2 (DADA2) is an autosomal recessive disease associated with a highly variable clinical presentation, including vasculitis, immunodeficiency, and hematologic manifestations, potentially progressing over time. The present study describes the long-term evolution of the immuno-hematological features and therapeutic challenge of two identical adult twin sisters affected by DADA2. The absence of plasmatic adenosine deaminase 2 (ADA2) activity in both twins suggested the diagnosis of DADA2, then confirmed by genetic analysis. Exon sequencing revealed a missense (p.Leu188Pro) mutation on the paternal *ADA2* allele. While, whole genome sequencing identified an unreported deletion (IVS6_IVS7del*) on the maternal allele predicted to produce a transcript missing exon 7. The patients experienced the disease onset during childhood with early strokes (Patient 1 at two years, Patient 2 at eight years of age), subsequently followed by other shared DADA2-associated features, including neutropenia, hypogammaglobulinemia, reduced switched memory B cells, inverted CD4:CD8 ratio, increased naïve T cells, reduced follicular regulatory T cells, the almost complete absence of NK cells, T-large granular cell leukemia, and osteoporosis. Disease evolution differed: clinical manifestations presented several years earlier and were more pronounced in Patient 1 than in Patient 2. Due to G-CSF refractory life-threatening neutropenia, Patient 1 successfully underwent an urgent hematopoietic stem cell transplantation (HSCT) from a 9/10 matched unrelated donor. Patient 2 experienced a similar, although delayed, disease evolution and is currently on anti-TNF therapy and anti-infectious prophylaxis. The unique cases confirmed that heterozygous patients with null ADA2 activity deserve deep investigation for possible structural variants on a single allele. Moreover, this report emphasizes the importance of timely recognizing DADA2 at the onset to allow adequate follow-up and detection of disease progression. Finally, the therapeutic management in these identical twins raises significant concerns as they share a similar phenotype, with a delayed but almost predictable disease evolution in one of them, who could benefit from a prompt definitive treatment like elective allogeneic HSCT. Additional data are required to assess whether the absence of enzymatic activity at diagnosis is associated with hematological involvement and is also predictive of bone marrow dysfunction, encouraging early HSCT to improve functional outcomes.

## Introduction

The deficiency of adenosine deaminase 2 (DADA2) is a monogenic autosomal recessive multisystem disease caused by loss-of-function mutations in the adenosine deaminase 2 (*ADA2*) gene encoding an adenosine deaminase enzyme that catalyzes the deamination of adenosine into inosine (1, 2). Monocytes and macrophages are the primary producers of ADA2 (3, 4). ADA2 loss causes increasing macrophage polarization towards the pro-inflammatory M1 phenotype at the expense of anti-inflammatory M2 macrophages (2). Consequently, the unbalanced macrophage polarization enhances the inflammatory processes and reduces tissue remodeling, ultimately leading to tissue damage in DADA2 patients.

Patients with DADA2 exhibit wide-ranging manifestations, including vasculitis, immunodeficiency, and hematologic disease. Bone marrow failure (BMF), pure red cell aplasia (PRCA), and severe neutropenia are life-threatening, disabling conditions that, together with vasculitis of the central nervous system and liver disease, represent the most severe manifestations (5–7). Deleterious mutations with absent or minimal residual enzymatic activity are associated with hematological manifestations such as BMF and PRCA (8). Although this correlation has been found in a large cohort of patients, it is challenging to predict the full-blown clinical phenotype based exclusively on the genotype. Indeed, the DADA2 phenotype represents a continuous spectrum of clinical manifestations rather than distinct categories. For instance, patients with vasculitis can develop anemia and leukopenia after years (8).

Clinical experience in DADA2 indicates that TNF inhibition ameliorates vasculitis but not cytopenias (8–10). Allogeneic hematopoietic stem cell transplantation (HSCT) is the treatment of choice for DADA2 patients presenting refractory cytopenias, with or without immunodeficiency and/or lymphoproliferation or malignancy (9, 11). Thus DADA2 patients with refractory cytopenias and BMF should be promptly candidate to HSCT, given the morbidity and mortality related to hemorrhage, iron overload, infections, and side effects of multiple immunosuppressive agents (8, 12).

It is unknown whether the pathogenesis of DADA2-associated hematological defects relates to autoinflammatory mechanisms or other factors. Given the variability of the disease, even among related patients (13–16), disease modifier genes and extrinsic factors in the bone marrow (BM) microenvironment might also contribute to the hematological development of DADA2 (8). Monozygotic twins are a unique model to characterize the evolution of genetic diseases. In this study, we describe the longitudinal follow-up of a couple of identical twin sisters diagnosed with DADA2 in adulthood and not previously treated with immunosuppressive drugs, representing the natural evolution of the disease.

## Case report

### Clinical findings

**Figure 1:**
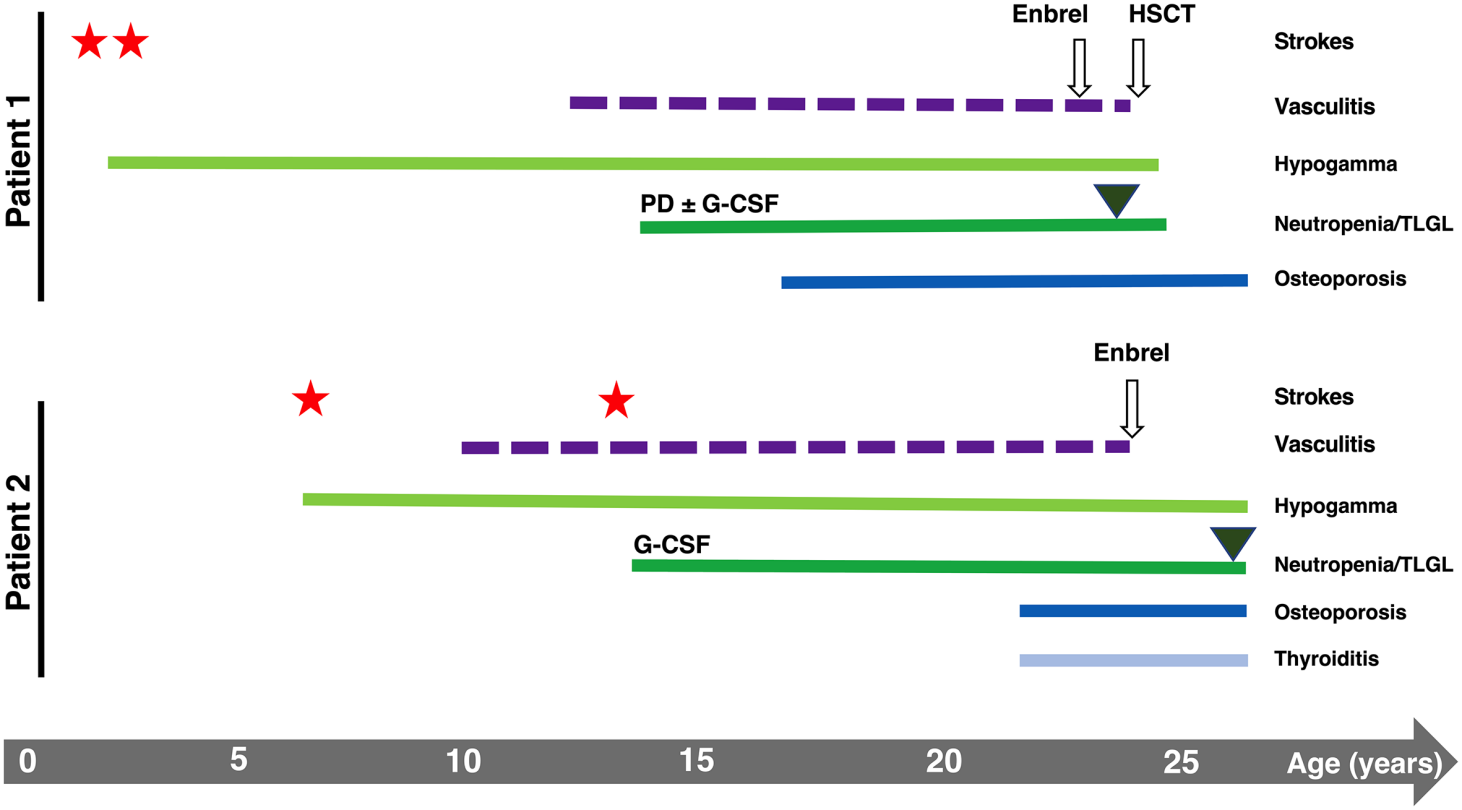
Graphical representation of the clinical history of P1 and P2. Stars and triangles represent strokes and T-LGL onset, respectively.

Two genetically identical twin sisters were recruited with suspected DADA2. P1 experienced two ischemic strokes at 2 and 3 years of age, lower limbs vasculitis (13 years), severe osteoporosis (17 years), and recurrent infections over life (recurrent abscesses, Varicella-Zoster virus (VZV) reactivation, *C. difficilis* enteritis, multiresistant *E. coli* sepsis, respiratory syncytial virus-B infection). P2 experienced two ischemic strokes at a later time than P1 (8 and 14 years), lower limbs vasculitis (10 years), severe osteoporosis (23 years), and recurrent infections (recurrent abscesses, two VZV reactivations, three herpes simplex virus-2 reactivations, genital candidiasis). Despite the similarities, the onset of clinical presentations was delayed in P2 (**Figure 1** **)**.

**Figure 2:**
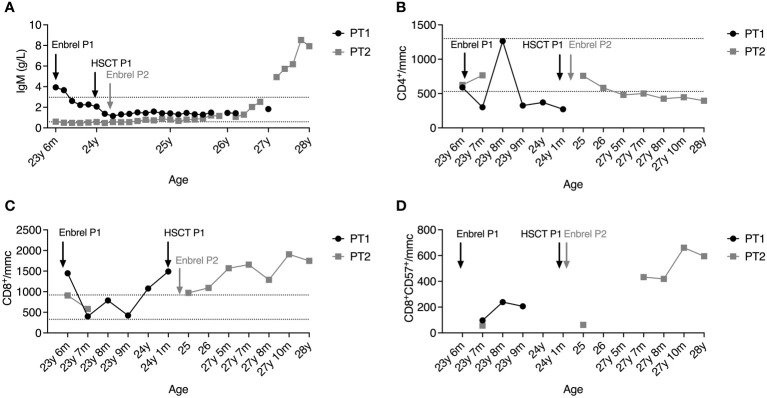
Plasmatic IgM levels **(A)**, absolute counts of CD4+ T cells **(B)** and CD8+ T cells **(C)**, and percentage of T-LGL **(D)** in the peripheral blood of P1 (black line) and P2 (grey line) after diagnosis. Dashed lines represent reference ranges in healthy controls.

Although the immunological features were similar in both patients, sharing the reduction of IgG and IgA, P1 presented at diagnosis with a high IgM titer (3.94 g/L). In contrast, up to one year from established diagnosis, P2 showed a defective IgM production, which raised constantly and gradually over three years, reaching a level of 7.93 g/L, independently of the persistence of low IgG, which required immunoglobulin supplementation (**Figure 2A**). In line with hypogammaglobulinemia, total and class-switched (CD27+IgD-IgM-) memory B cells were below the normal range for patients’ age (**Table S1**), as typically reported in DADA2 patients (17, 18). NK cells were virtually absent (< 0.5% of lymphocytes). The CD4:CD8 ratio was inverted at diagnosis, more markedly in P1, demonstrating a progressive worsening over time in both patients due to CD4+ T cell lymphopenia (**Figure 2B**) and expansion of CD8+ T cells (**Figure 2C**). Recent thymic emigrants (CD31+CD4+ T cells) were well represented in P1 and P2 despite adult age (**Table S2**). Similarly, naïve (CD45RA+CD62L+) were preponderant, while total memory (CD45RO+), central memory (CD27+CD45RA-), and effector memory (CD27-CD45RA-) T cells were below normal values (**Table S2**), in line with the immunological phenotype described in DADA2 (18). Follicular helper (CXCR5+CD45RA-) CD4+ T cell frequencies were within the normal range, while follicular regulatory (CXCR5+FoxP3+) T cells were reduced (**Table S2**). The CD8+ T cell compartment displayed a similar distribution where naïve CD8+ cells prevailed, but the effector memory subset (CCR7-CD27-CD45RA+) was increased (**Table S2**).

Both patients developed hematological manifestations (**Figure 1** **)**. They experienced the onset of severe neutropenia at the same time when they were 14 years old, manifesting vulvar infection (P1) or abscess (P2) and oral aphthous lesions (both) during the months preceding the diagnosis. Moreover, both twins showed neutrophil counts below 100 cells/mmc associated only with a transient positivity of IgM anti-neutrophil antibodies (with negative IgG). P1’s BM aspirate showed a reduction in the maturation rate of neutrophils, and BM biopsy showed polymorphic lymphoid infiltrates (mainly CD4+ T cells). For P2, the only available BM aspirate showed a maturation delay of myeloid progenitors. P1 showed severe steroid-dependent neutropenia progressively unresponsive to increasing doses of granulocyte-colony stimulating factor (G-CSF) associated with T-large granular lymphocytes (T-LGL) leukemia with BM involvement, requiring HSCT when she was 24 years old. P2 presented less severe neutropenia responsive to chronic G-CSF administration, never treated with steroids.

At 26 years of age, P2 also presented a T-LGL component on the peripheral blood (PB), and circulating T lymphocytes harboring clonal TCR (beta and gamma) have been identified in the absence of blasts (data not shown). The progressive T-LGL expansion in the PB of both patients may explain the marked inversion of the CD4:CD8 ratio. We then examined the T-LGL subset in the BM of P1 before HSCT. Histological examination showed hypercellularity represented by the 50-60% of interstitial CD8+ T lymphocytes of small-intermediate size partially positive for granzyme B. Biopsies of P2’s BM analyzed at 23 and 27 years of age revealed an infiltrate of lymphocytes gradually increasing from 30% to 40% of total BM cells, with a consistent component of small CD8+ T lymphocytes classifiable as T-LGL. Phenotypically, the CD8+CD57+ T-cell subset expanded from 11.8% to 19% of total BM lymphocytes, mirroring the expansion observed in the PB (**Figure 2D**).

Finally, the functional immunological characterization showed a normal *in vitro* response to polyclonal mitogens in both patients. In contrast, the response to Candida inactivated spores was absent in P1 and weakly positive in P2, despite previous genital infection (data not shown). The levels of cytokines in plasma and those produced *in vitro* from peripheral blood mononuclear cells were normal (data not shown).

### Diagnostic assessment

**Figure 3:**
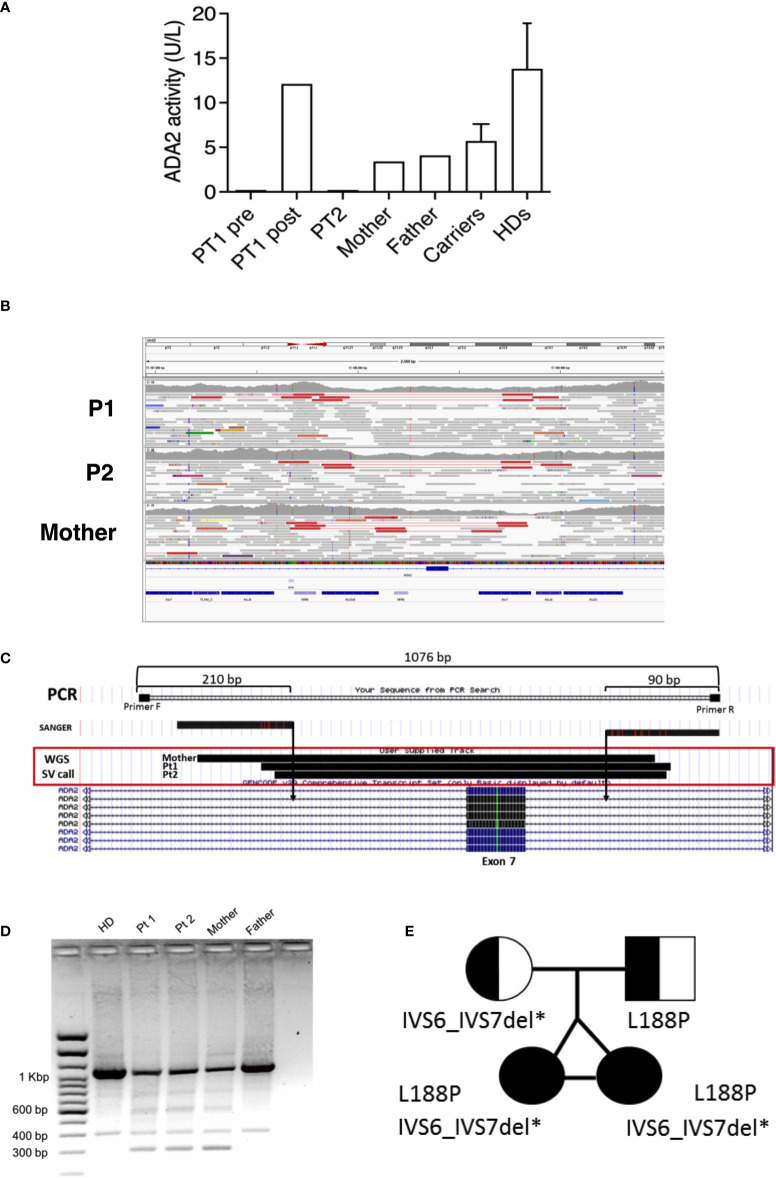
**(A)** ADA2 plasmatic activity in P1 (before and after HSCT), P2, parents, carriers (n= 46), and healthy controls (n=27). **(B)** Visualization of mapped reads (as pairs) with Integrative Genomics Viewer (IGV) around the ADA2 exon 7. The tracks related to P1, P2, and the mother are depicted from top to bottom. On the top of each track, pairs (in red) spot the deletion event. Reads colored in grey represent properly mapped paired-end reads. **(C)** Graphical representation of PCR amplification used for validating the deletion. **(D)** Short amplicons of 300 bp visualized by agarose gel electrophoresis in P1, P2, and the mother. **(E)** Pedigree of the affected twin sisters, compound heterozygous for the missense mutation L188P and IVS6_IVS7 deletion in the *ADA2* gene.

P1 and P2 underwent next-generation (targeted) sequencing analysis at 22 years of age and resulted in heterozygous for a paternal missense mutation (c.563T>C; p.Leu188Pro) in the *ADA2* gene primary transcript (NM_001282225). No second pathogenic variant could be identified in the coding sequence even though plasma ADA2 activity was completely absent in patients and reduced in parents, compatible with a carrier status for both of them (**Figure 3A**). Considering the clinical history of the twins and the levels of enzymatic activity of the whole family, we suspected the mother was a carrier of an *ADA2* gene variant. Thus, a whole-genome sequencing analysis (WGS) was performed on the patients and the mother to investigate the presence of other possible disease-causing variants. After WGS read quality control, trimming, and alignment against the GRCh38 reference genome, variant calling of single nucleotide variants (SNVs) and small insertions/deletions (INDELs) was performed using the Genome Analysis Toolkit (GATK) (19) in the region of the ADA2 gene both in the mother and the daughters. Using SnpEff (20), we performed the annotation of these variants, with the result that none of them presented pathogenic effects and were inherited from the mother. Thus, on the same WGS data, we explored the possible presence of large structural variants using Delly (21), which revealed a heterozygous deletion (chr22: 17187840 and chr22:17188716) causing the exon 7 excision both in the mother and daughters (**Figures 3B, C**). This structural variant was confirmed by amplifying the interested region using flanked primers, which revealed the presence of a short amplicon of 300 bp in the patients and the mother, but not in the father or a healthy control (**Figure 3D**). Sanger sequencing of this 300 bp amplicon confirmed the exon 7 deletion. Thus, the patients were compound heterozygous for the p.Leu188Pro missense mutation (in the ADA2 putative receptor binding domain) and exon 7 deletion (disrupting the catalytic domain of the protein) (**Figure 3E**). AmpFlSTR^®^ Identifiler^®^ Plus, PCR Amplification Kit short tandem repeat (STR) multiplex assay, amplifying 15 tetranucleotide repeat loci and the Amelogenin in single PCR amplification showed complete overlap of all loci of P1 and P2, confirming that the patients were genetically identical twins.

### Therapeutic intervention

The therapeutic approach for both patients consisted of etanercept, G-CSF, and anti-infectious prophylaxis (substitutive immunoglobulins, acyclovir, fluconazole). Due to the worsening of steroid-dependent neutropenia associated with immunodeficiency, P1 was transplanted with BM HSC from a matched unrelated donor (MUD) 9/10 HLA-matched when at 24 years of age. She received a treosulfan-thiotepa-fludarabine-based conditioning regimen before transplantation. The graft consisted of 2.45 x 10^8 total nucleated cells (TNC)/kg, 3.02 x 10^6 CD34+/kg, 48.34 x 10^6 CD3+/kg. Rituximab, ATG, methotrexate, and cyclosporine A were administered as graft versus host disease (GVHD) prophylaxis. Neutrophil and platelet engraftment occurred on days +24 and +22, respectively. Complete donor chimerism was detected on day +33. The patient did not experience GVHD, and the only post-HSCT complication was febrile neutropenia. P1 no longer experienced stroke recurrences, and the immunological phenotype normalized, accompanied by the complete recovery of plasma ADA2 activity (**Figure 2A**). At present, she is completely independent of any immunosuppression and anti-infectious prophylaxis.

Up to now, P2 has been under G-CSF for 12 years, and she is still responsive. She is a candidate for life-long immunosuppression and anti-infectious prophylaxis to avoid vascular or infectious events recurrences.

### Clinician and patient-assessed outcomes

P1 is completely cured four years after transplant, experiencing everyday life. In contrast, P2 is currently on multiple medications (G-CSF, etanercept, immunoglobulins, anti-fungal, and anti-viral prophylaxis) to control the clinical manifestations of the disease and prevent infectious complications. Indeed she experienced neither stroke recurrence, vasculitis, nor systemic inflammation. Nevertheless, she requires a regular follow-up, considering the evolving hematological phenotype with T-LGL expansion, refractory to anti-TNF therapy, and chronic disease burden. P2 is closely monitored and fully conscious of the possible evolution of her disease, as occurred in her sister’s case. The prevision of the disease course remains uncertain, and the perspective of definitive therapy is a currently unmet clinical need.

## Discussion

The two patients’ clinical history recapitulated the progression of DADA2 over the course of life. The patients manifested the DADA2 three main clinical features: autoinflammation, immunodeficiency, and hematological alterations. The two cases described herein underline critical issues in the diagnosis, follow-up, and therapeutic management of patients with DADA2. P1 and P2 were misdiagnosed for years, as DADA2 was still unknown when they experienced the onset of the disease. Thus, their clinical evolution from infancy to adulthood is an example of DADA2 natural history in the absence of targeted therapy, as immunosuppression was started in their twenties after genetic diagnosis. Although the enzymatic assay promptly revealed a complete loss of ADA2 activity, the definitive molecular diagnosis has been further delayed due to the impossibility of identifying structural variants by targeted sequencing, which only detected a missense mutation on the paternal allele. However, the absence of ADA2 enzymatic activity suggested the presence of two deleterious mutations prompting WGS analysis, which revealed a deletion on the maternal allele, thus confirming the diagnosis of DADA2. Recent literature recognized an increasing number of structural variants in the region encompassing exon 7 (22), probably related to repetitive elements (Alu sequences) that make this region more prone to indels. Hence, heterozygous patients exhibiting the absence of ADA2 enzymatic activity deserve additional investigations to identify another pathogenic variant on the other allele, which is valuable information for patients’ follow-up and familial counseling.

The intrafamilial variability and the heterogeneous clinical phenotype among unrelated patients carrying the same mutations are well-known characteristics of the DADA2 (13, 14, 23). Reports on twins couples revealed that epigenetic differences among twins exist (24). Cells with the same DNA sequence can display a unique phenotype due to the suppression or induction of gene expression. Several epigenetic mechanisms have been reported. DNA methylation, responsible for gene repression, is highly influenced by the environment if exposure to different conditions, such as infections, occurs over time (24–26). A large cohort study of healthy twins suggests that environment combines with the genetic background, making a relevant contribution to the development and evolution of the immune system throughout life (27). Our twin patients have been living together until now and share similar habits, but their infectious history might differ. Similarly, chromatin remodeling mediated by histone acetylation is indistinguishable at an early age but can diverge significantly over time (24), possibly explaining why phenotypes of identical twins may diverge during their lifetime. Lastly, differential expression of post-transcriptional small-noncoding RNAs (miRNAs) among twins have been described as a key element in modifying the expression of gene related to some diseases justifying the different phenotype of monozygotic twins (28). Studies on twins affected by monogenic inborn errors of immunity are scarce or limited to case reports describing a certain grade of divergence in the clinical evolution of identical twins (29). In this case report, we describe the evolution of two identical adult twins sharing the same genotype and genetic background living together and exposed to common environmental factors since birth. All these factors make these patients ideal for gaining insights into the evolution of DADA2 over time. Overall, the patients experienced almost the same clinical manifestations, particularly the hematological ones, with latency in P2 compared to P1.

It has been reported that the mutations that abrogate ADA2 activity have the most deleterious effect on hematopoiesis (8, 30). Indeed, the genotype of P1 and P2 (compound heterozygous for a missense mutation with predicted minimal enzymatic activity (<3%) and a genomic deletion leading to complete absence of enzymatic activity) was predictive of PRCA and BMF in these patients. Therefore, it cannot be excluded that adult patients with severe hematological manifestations (neutropenia, BMF, PRCA) received allogeneic HSCT without a genetic diagnosis of DADA2 (31). These observations suggest P2 might risk developing a condition similar to P1. Indeed, the close monitoring of P2 allowed identifying the T-LGL in BM and PB, a condition reported in patients affected by DADA2 (32, 33).

At present, P2 does not yet meet the criteria for therapeutic intervention, justified only in the case of T-LGL-induced severe neutropenia, moderate neutropenia with recurrent infections, transfusion-dependent anemia, and autoimmune conditions requiring therapy (34). However, we should consider patient-specific features. Although P2 has been treated with G-CSF for more than ten years for pre-existing neutropenia and more recently with anti-TNF therapy (which increases the rate of immunosuppression), she still exhibits immunodeficiency and a genetic predisposition to develop severe hematological manifestations related to BM dysfunction. These data suggest that additional immunosuppression (such as first-line methotrexate) to treat T-LGL would likely worsen the risk of infections without resolving the underlying G-CSF-dependent neutropenia.

Finally, considering the twin sisters’ clinical history and identical genetic background, the critical question of whether P2 would benefit from an early HSCT still remains. Despite the described benefits of transplantation on the disease manifestations, a genetic diagnosis of DADA2 is not an indication for HSCT upfront due to the risks related to the procedure (drug toxicity, graft versus host disease, transplant-related mortality). On the other hand, recent publications report refractory neutropenia, BMF, PRCA, and severe immunodeficiency as the main indications in HSCT (9, 11, 12). Based on the disease evolution of the sister, this patient would probably benefit from transplantation. The standard indication and timing definition for HSCT in DADA2 deserve further discussion and outcome studies.

## Conclusions

This report highlights the importance of a timely diagnosis in DADA2 suggested by the reduced levels of plasma ADA2 activity. Patients heterozygous for missense mutations with minimal or absent ADA2 activity should be considered affected and treated, even without molecular confirmation, which sometimes requires further detailed and time-spending studies. However, these patients deserve further investigation for possible structural variants on the other allele since identifying pathogenic mutation may anticipate some phenotypical features. This allows setting up an appropriate follow-up to recognize subtle changes suggesting disease progression and support familial counseling.

The therapeutic management of these identical twins raised concerns as, albeit they share a similar but delayed phenotype, disease evolution could be predictable in one of them, thus benefiting from elective allogeneic HSCT. Additional data are required to assess whether the absence of enzymatic activity associated with deleterious mutations can predict the risk of hematological manifestations and the progression to BM dysfunction, encouraging early HSCT to improve functional and clinical outcomes limiting disease evolution. Likewise, further studies on these patients may reveal prognostic markers for the progression of the hematological disease to anticipate the indication for definitive therapy, lowering morbidity and mortality related to HSCT in older age.

In conclusion, it is crucial to increase the physicians’ awareness on DADA2, rising critical questions on the best approach to diagnose and treat patients. It will also be essential to consider, in selected cases, to anticipate curative treatment preceding the progression of the disease.

## Data availability statement

The datasets presented in this study can be found in online repositories. The name of the repository and accession number can be found below: European Nucleotide Archive (https://www.ebi.ac.uk/ena/browser/home); accession number PRJEB52333.

## Ethics statement

The studies involving human participants were reviewed and approved by San Raffaele Hospital Ethical Committee. The patients/participants provided their written informed consent to participate in this study. Written informed consent was obtained from the individual(s) for the publication of any potentially identifiable images or data included in this article.

## Author contributions

AM and AA conceived and supervised the study and critically revised the manuscript. FB designed the study; FB and MPC wrote the manuscript; FB, MPC, MZo, and IB acquired, analyzed, and interpreted data. CS, RM, TJ, and GF provided immuno-hematological data. FB, MPC, and SM provided patient material. MZo, IB, MB, IM, MZa provided and analyzed genetic data. LB and MP evaluated pathology. MH provided enzyme activity data. VC, MEB, FT, MM, FG, MD, DC, FF, GC, GP, FS, SB, PR-Q, FP, MG, JP, and FC clinically followed the patients and critically revised the manuscript. All authors contributed to the article and approved the submitted version.

## Funding

This work was funded by Fondazione Telethon (SR-Tiget Core Grant, Tele21-A5) to AA and AM; E-rare EUROCID project, Italian Ministry of Health (Progetto rete IDEA RCR-2020-23670068_001), the Jeffrey Modell Foundation, Else Kröner Fresenius Prize for Medical Research 2020 to AA.

## Acknowledgments

The authors thank the patients, family members, and staff from all clinical units participating in the study. IRCCS Istituto Giannina Gaslini and IRCCS San Raffaele Hospital are part of the European Reference Network for Rare Immunodeficiency, Autoinflammatory and Autoimmune Diseases, ERN-RITA (Project ID 739543). The centers involved are part of the Italian Onco-Hematology Association (AIEOP). San Raffaele Hospital is part of the Inborn Error Working Party of The European Society for Blood and Marrow Transplantation (EBMT).

## Conflict of interest

The authors declare that the research was conducted in the absence of any commercial or financial relationships that could be construed as a potential conflict of interest.

## Publisher’s note

All claims expressed in this article are solely those of the authors and do not necessarily represent those of their affiliated organizations, or those of the publisher, the editors and the reviewers. Any product that may be evaluated in this article, or claim that may be made by its manufacturer, is not guaranteed or endorsed by the publisher.
