# Supplementary material for: Case Report: Consistent disease manifestations with a staggered time course in two identical twins affected by adenosine deaminase 2 deficiency
Source: Front Immunol. 2022 Sep 29;13:910021. doi: 10.3389/fimmu.2022.910021 (PMC9557171; doi:10.3389/fimmu.2022.910021)
Supplement: Supplementary file 1 [file Table_1.docx]

**Supplementary Table 1. Naive and memory B-cell subsets**

|  | **P1** | | **P2** | | **Adult** |
| --- | --- | --- | --- | --- | --- |
|  | **Before**  **HSCT** | **Post-HSCT**  **(5 m)** | **Before**  **Anti-TNF** | **Under**  **Anti-TNF (5m)** | **Normal Range** |
| **% of CD19^+^** |  |  |  |  |  |
| CD21^low^ CD38^low^ (Autoreactive) | 2.1 | 0.4 | 0.6 | **9.7** | 4.3 ± 2.2 |
| CD24^bright^ CD38^bright^ (Transitional) | 12.2 | 50.8 | 10.4 | 2.6 | 5.2 ± 4.7 |
| CD24^bright^ CD38^low^ (Activated) | 13.2 | 0.0 | 11.5 | 9.7 | 24.7 ± 13.4 |
| CD24^low^ CD38^low^ (Mature) | 65.3 | 27.4 | 73.1 | 70.4 | 58.9 ± 14.8 |
| CD24^neg^ CD38^bright^ | 0.7 | 10.9 | 1.2 | 11.8 | 4.9 ± 5.5 |
| CD27^-^ (Naive) | **84.7 ↑** | 97.7 | **88.5 ↑** | **78.7 ↑** | 62.5 ± 15.6 |
| IgD^+^ IgM^+^ | 83.6 | 95.1 | 68.1 | 95.2 | 67.5 ± 14.3 |
| CD27^+^ (Memory) | 14.2 | 2.3 | 10.4 | 21.3 | 35,2 ± 15,5 |
| IgD^-^ IgM^-^ (Switched) | **17.1 ↓** | 6.3 | **5.4 ↓** | **8.2 ↓** | 49.6 ± 5.8 |
| CD24^neg^ CD38^bright^ | 0.0 | 59.0 | 3.4 | 35.0 | 6.2 ± 5.2 |

**Supplementary Table 2. Naive and memory T-cell subsets**

|  | **P1** | | | **P2** | | | **Adult** |
| --- | --- | --- | --- | --- | --- | --- | --- |
|  | **Before**  **HSCT** | **Post**  **HSCT**  **(5 m)** | **Post**  **HSCT**  **(4 y)** | **Before**  **Anti-TNF** | **Under**  **Anti-TNF**  **(5 m)** | **Under**  **Anti-TNF**  **(4 y)** | **Normal**  **Range** |
| **% of CD45^+^** |  |  |  |  |  |  |  |
| CD3^+^ | **94.9 ↑** | 65.6 | 78.1 | **88.5 ↑** | **85.7 ↑** | **96.8 ↑** | 78.8±6.6 |
| Double Negative TCRαβ^+^CD3+CD4-CD8- | 0.4 | 0.3 | 1.0 | 0.9 | 1.0 | 0.2 | 2.2±1.0 |
| CD3^+^ CD4^+^ | **31.1 ↓** | 73.4 | 62.4 | **37.3 ↓** | 52.9 | **16.9 ↓** | 53.5±10.9 |
| CD3^+^ CD8^+^ | **65.9 ↑** | 16.4 | 32.9 | **55.1 ↑** | 42.7 | **81.8 ↑** | 36.8±11.2 |
| **% of CD45^+^ CD3^+^CD4^+^** |  |  |  |  |  |  |  |
| CD27^+^ CD45RA^+^ (Naive) | **85.3 ↑** | 55.6 | 74.1 | **67.2 ↑** | **93.7 ↑** | **85.5 ↑** | 56.3±10.4 |
| CD27^+^ CD45RA^-^ (Central memory) | **12.7 ↓** | 33.0 | 23.3 | 27.9 | **4.7 ↓** | **10.2 ↓** | 33.6±7.3 |
| CD27^-^ CD45RA^-^ (Effector memory) | **1.3** **↓** | 10.9 | 2.6 | **4.7** | **0.3 ↓** | **1.8 ↓** | 8.2±3.6 |
| CD27^-^ CD45RA^+^ (TEMRA) | 0.7 | 0.4 | 0.1 | 0.2 | 1.3 | 2.6 | 3.6±1.9 |
| CD31^+^ CD45RA^+^ (Recent thymic emigrants) | **63.7 ↑** | 51.8 | 64.0 | **46.9 ↑** | **55.1 ↑** | **47.1 ↑** | 32.9±9.1 |
| CXCR5+CD45RA-(T follicular helper) | 15.5 | nd | 12.8 | nd | nd | 22.4 | 16.8±5.6 |
| CXCR5+FoxP3+ (T follicular regulatory) | **0.2↓** | nd | 1.7 | nd | nd | **0.47↓** | 2.38±1.9 |
| **% of CD45^+^ CD3^+^CD8^+^** |  |  |  |  |  |  |  |
| CCR7^+^ CD27^+^ CD45RA^+^ (Naive) | 19.8 | 74.5 | 78.3 | 32.6 | 43.7 | 5.4 | 52.9±14.7 |
| CCR7^-^ CD27^+^ CD45RA^-^ (Central memory) | **0.1 ↓** | 9.4 | 10.8 | **0.2 ↓** | **0.2 ↓** | 0.1 | 3.7±2.1 |
| CCR7^-^ CD27^-^ CD45RA^-^ (Effector memory) | **0.0 ↓** | 5.1 | 2.0 | **0.1 ↓** | **0.0 ↓** | 0.0 | 17.4±10.9 |
| CCR7^-^ CD27^-^ CD45RA^+^ (TEMRA) | **70.8 ↑** | 1.8 | 2.8 | 19.8 | 24.5 | **85.3 ↑** | 14.1±10.9 |
